# Altered oxidant and antioxidant levels are associated with vascular stiffness and diabetic kidney disease in type 1 diabetes after exposure to acute and chronic hyperglycemia

**DOI:** 10.1186/s12933-024-02427-4

**Published:** 2024-09-28

**Authors:** Krishna Adeshara, Elyse Di Marco, Marco Bordino, Daniel Gordin, Luciano Bernardi, Mark E Cooper, Per-Henrik Groop, on behalf of the FinnDiane Study Group

**Affiliations:** 1 Folkhälsan Research Center, Biomedicum Helsinki, Haartmaninkatu 8, FIN-00290 Helsinki, Finland; 2 Research Program for Clinical and Molecular Metabolism, University of Helsinki, Helsinki, Finland; 3 Department of Diabetes, Central Clinical School, Monash University, Melbourne, VIC Australia; 4 Department of Nephrology, University of Helsinki and Helsinki University Hospital, Helsinki, Finland; 5 Minerva Foundation Institute for Medical Research, Helsinki, Finland; 6 Joslin Diabetes Center, Harvard Medical School, Boston, MA USA; 7 Baker Heart and Diabetes Institute, Melbourne, VIC Australia

**Keywords:** Oxidative stress, Antioxidant capacity, Diabetes, Hyperglycemia, Arterial stiffness, Diabetic kidney disease

## Abstract

**Background:**

Hyperglycemia-induced oxidative stress is a well-established pathological mediator of vascular complications in diabetes. We assessed plasma oxidant and antioxidant levels in response to acute and chronic hyperglycemia in relation to vascular stiffness and varying degrees of kidney disease in type 1 diabetes individuals.

**Methods:**

The acute hyperglycemia study included 22 type 1 diabetic individuals with normal albumin excretion rate (AER) and 13 non-diabetic controls. These individuals received an acute glucose challenge during a 120-minute hyperglycemic clamp. The chronic hyperglycemia study included 118 type 1 diabetic individuals with chronically low (*n* = 60) or high (*n* = 58) HbA1c concentrations and varying degrees of diabetic kidney disease (DKD) classified as normal, moderate, or severe albuminuria (AER). Levels of malondialdehyde (MDA), reactive oxygen metabolites (ROMs), total antioxidant capacity (TAC), biological antioxidant potential (BAP) and superoxide dismutase (SOD) were measured from plasma or serum samples in the FinnDiane study.

**Results:**

Levels of MDA (*p* < 0.01) and ROMs (*p* < 0.01) were elevated in type 1 diabetes individuals compared to non-diabetic controls at baseline. Acute hyperglycemia further increased MDA levels (*p* < 0.05) and sustained the elevation of ROMs in type 1 diabetes individuals. Acute hyperglycemic challenge impaired TAC in both non-diabetic (*p* < 0.05) and type 1 diabetes (*p* < 0.01) individuals compared to baseline whereas BAP was increased (*p* < 0.05) with no difference observed in non-diabetic controls. There was a positive association between high circulating MDA and AIx (r2 = 0.611, *p* = 0.05), and between delta ROMs and delta AIx (r2 = 0.955, *p* = 0.014) in combined analysis of individuals with type 1 diabetes and non-diabetic controls. Type 1 diabetes individuals with varying status of DKD, showed elevated levels of ROMs in those with high HbA1c compared to their counterpart with low HbA1c (*p* < 0.05). Individuals with severe albuminuria showed elevated ROM levels (*p* < 0.01) and depressed antioxidant capacity (*p* < 0.01) compared to those with normal AER of comparable HbA1c concentrations.

**Conclusions:**

Biomarkers of oxidative stress are associated with vascular stiffness and DKD following acute and chronic hyperglycemic exposure and may provide added value to HbA1c in understanding disease pathology, predicting risk and assessing the status of secondary complications of type 1 diabetes.

**Supplementary Information:**

The online version contains supplementary material available at 10.1186/s12933-024-02427-4.

## Introduction

Extensive experimental and clinical evidence supports a central role of oxidative stress in the development and progression of diabetic complications such as arterial stiffness, atherosclerosis and diabetic kidney disease (DKD) [1]. Chronic and acute exposure to hyperglycemia increases oxidative stress and excessive production of reactive oxygen species (ROS) through enzymatic and non-enzymatic sources. Excessive ROS increases inflammatory and adhesion factors that, in turn, promote cell migration to atherosclerotic lesions and increase plaque instability [2]. Poor glycemic control characterized by acute fluctuations in blood glucose is associated with activation of oxidative stress in individuals with type 1 [3] and type 2 diabetes [4]. In a population-based study, subjects with impaired (≥ 6.1 to < 7.0 mmol/l) or abnormal (≥ 7.0 mmol/l) fasting glucose showed elevated levels of lipid peroxidation products and suppressed levels of the antioxidant enzyme substrate glutathione compared to people with normal fasting glucose [5]. We have previously showed that an acute hyperglycemic challenge increases plasma antioxidant superoxide dismutase (SOD) levels in healthy non-diabetic controls but not in individuals with type 1 diabetes [6]. Unrestrained oxidant production promotes endothelial cell dysfunction, inactivation of nitric oxide, pro-inflammatory cytokines secretion [7] and direct damage to macromolecules. Acute hyperglycemia increased risk of atherothrombotic events, fibrinolytic imbalance, and endothelial dysfunction in both diabetic and non-diabetic subjects [8, 9]. Increased levels of nitrotyrosine as a marker of oxidative stress was observed in non-diabetic subjects during an acute hyperglycemic clamp [10]. However, the response to oxidative stress upon acute or chronic hyperglycemia is not yet known in type 1 diabetes.

Arterial stiffness is strongly associated with the development and progression of microvascular complications of diabetes, in particular DKD [11]. Pathological generation of ROS promotes podocyte injury, tubular damage, and mesangial cell extracellular matrix deposition [12] leading to albuminuria and ultimately kidney failure. Targeted genetic deletion of the principal ROS-producing enzymes in the kidney, nicotinamide adenine dinucleotide phosphate (NADPH) oxidase isoform 4 (NOX4), was shown to attenuate diabetes-induced albuminuria in mice [1]. Similarly, polymorphisms in the *CYBA* gene that encodes the p22phox regulatory subunit of NOX was associated with elevated systemic oxidative stress and increased incidence or progression of DKD in individuals with type 1 diabetes [13]. Chronic hyperglycemia assessed from HbA1c increases determinants of oxidative stress and risk of vascular complications in type 2 diabetes [14]. Importantly, HbA1c variability is predictive of incident microalbuminuria and progression of kidney disease in type 1 diabetes [15]. Despite rigorous efforts to improve blood glucose control, heterogeneity in kidney outcomes still exists. In the present study, we aimed, (1) to examine the oxidant and antioxidant responses in relation to an acute hyperglycemic challenge and their relationship with vascular stiffness; (2) to assess differences in oxidant and antioxidant levels in relation to chronic hyperglycemia with varying degrees of DKD in individuals with type 1 diabetes.

## Methods

### Study subjects and protocol

Adults with type 1 diabetes from the Finnish Diabetic Nephropathy (FinnDiane) Study were included in this study. Type 1 diabetes is defined as age at diabetes onset < 40 years and permanent insulin treatment started within one year after the diagnosis. Urinary albumin excretion rate (AER) was determined at the baseline FinnDiane visit and regularly during follow-up from at least two timed overnight or 24-h urine collections. Normal AER was defined as AER < 20 µg/min or < 30 mg/24 h, moderate albuminuria as AER ≥ 20 and < 200 µg/min or ≥ 30 and < 300 mg/24 h, severe albuminuria as AER ≥ 200 µg/min or ≥ 300 mg/24 h. Blood samples as well as specimens of urine were collected for laboratory measurements including HbA1c, lipids, creatinine, and urinary albumin excretion. This study was conducted in two parts: (1) Acute hyperglycemia study, (2) Chronic hyperglycemia study with varying status of DKD.

#### Acute hyperglycemia study

This study included 22 type 1 diabetic individuals with normal AER and 13 non-diabetic controls. The age of the participants varied between 18 and 40 years. Exclusion criteria were smoking, hypertension, arrhythmias, any medical treatment (except insulin), acute infections and diabetic complications. A total of 52 men participants were invited to take part in the study. However, 17 participants were excluded due to exclusion criteria. The information about the absence of diabetic retinopathy and normal ECG recordings were collected from medical records. The procedure for the hyperglycemic clamp has been described previously (6; 11). Briefly, individuals with type 1 diabetes received a bolus injection of 0.25 g/kg glucose (50% solution) followed by a 20% glucose infusion to achieve a steady-state plasma glucose concentration of 15 mmol/L for 120 min. Prior to the bolus injection, non-diabetic controls were given a 25 µg bolus (Sandostatin^®^, Novartis, Espoo, Finland) followed by a 0.5 µg/min infusion of a somatostatin analogue in order to suppress the endogenous insulin secretion. The infusion of the somatostatin analogue was interrupted at the end of the clamp. Blood samples for the analysis of oxidative stress markers were drawn at baseline (prior to hyperglycemia) and after 120 min of hyperglycemic clamp. Plasma or serum was separated and stored at -80 °C until further use.

**Sample size and power calculation**

Power calculation regarding the acute hyperglycemia study was carried out by using the general linear model with repeated measures test with IBM SPSS Statistics 29.0 (IBM Corporation, Somers, NY, USA). The significance threshold was set at α = 0.05, and the effect size was described in terms of partial Eta squared. The observed power values were as follows: 48% for malondialdehyde (MDA) (partial Eta squared = 0.108, *n* = 33), 58% for reactive oxygen metabolites (ROM) (partial Eta squared = 0.173, *n* = 25), 99% for total antioxidant capacity (TAC) (partial Eta squared = 0.371, *n* = 35), 12% for antioxidants (partial Eta squared = 0.020, *n* = 33), and 7% for superoxide dismutase (SOD) (partial Eta squared = 0.671, *n* = 35).

**Arterial stiffness measurements**

Arterial stiffness was assessed by applanation tonometry (SphygmoCor; Atcor Medical, Sydney, Australia) at baseline and 120 min of hyperglycemia. The pulse wave velocity (PWV) was recorded from the radial artery of the right arm with a high-fidelity micromanometer (SPC-301; Millar Instruments, Houston, TX, USA). To measure arterial stiffness in large (aortic) and intermediate-sized (brachial) arteries, carotid-femoral (aortic) and carotid-radial (brachial) PWV pressure waveforms were recorded sequentially at both the carotid, femoral and radial artery. With a simultaneous ECG recording of the R wave as a reference frame, the system software calculated the PWV [11]. The difference in carotid to femoral and carotid to radial path length was estimated from the distance from the sternal notch to the femoral and carotid palpable pulse. The augmentation index (AIx) is an indirect measure of arterial stiffness in the small arteries, calculated by dividing augmentation pressure with pulse pressure and expressed as a percentage. The average of three consecutive readings, each consisting of at least 20 sequentially recorded waveforms were used for the analyses. Additionally, in the healthy volunteers, an additional measurement was made after the somatostatin analogue infusion, but before the glucose bolus. All measurements were performed by a single operator (DG).

**Biochemical analysis of oxidative stress markers**

Levels of malondialdehyde (MDA), a lipid peroxidation product were measured in plasma using Cayman’s thiobarbituric acid reactive substances (TBARS) assay kit (#10009055, Cayman Chemicals, Ann Arbor, MI, USA), according to the manufacturer’s instructions. The concentration of MDA was calculated against an MDA standard (0–50µM) and expressed as MDA (µM). Plasma levels of reactive oxygen metabolites (ROMs) were measured using a commercially available kit (d-ROMs test; Diacron International, GR, Italy). The results were expressed as Caratelli Units (U.CARR.), and 1 U.CARR. is equivalent to 0.08 mg/100 mL of hydrogen peroxide [16, 17]. The total antioxidant capacity (TAC) was measured according to manufacturer’s instructions using Cayman’s Antioxidant Assay Kit (#709001, Cayman Chemicals, Ann Arbor, MI, USA). Trolox, a tocopherol analogue was used as standard to calculate the TAC of plasma samples and expressed as mM Trolox equivalents. Biological antioxidant potential (BAP) was assayed using a commercially available kit (BAP test; Diacron International, GR, Italy) and results were expressed as micromoles (µm) of reduced iron per liter of plasma [16, 17]. The concentration of superoxide dismutase (SOD) in serum samples was determined using a commercially available kit (Cayman Chemicals, Ann Arbor, MI, USA), as reported previously [6]. The activity of SOD was calculated from SOD standard (0-0.05U/mL) and expressed as U/mL.

#### Chronic hyperglycemia and diabetic kidney disease study

This study included 118 type 1 diabetic individuals with available HbA1c measurements for at least 5 years before sample collection. The mean HbA1c values were calculated from 15 ± 6 (mean ± SD) measurements per individual. Based on the distribution of mean HbA1c values, low HbA1c corresponds to 5.8 − 7.3% [40–56 mmol/mol] and high HbA1c to 10.2 − 12.5% [88–113 mmol/mol]. Individuals were stratified based on varying status of DKD along with low or high HbA1c, (1) Normal AER with low HbA1c, *n* = 20; (2) Normal AER with high HbA1c, *n* = 20; (3) moderate albuminuria with low HbA1c, *n* = 20; (4) moderate albuminuria with high HbA1c, *n* = 18; (5) severe albuminuria with low HbA1c, *n* = 20; (6) severe albuminuria with high HbA1c, *n* = 20. The information regarding medication (renin-angiotensin system blockers or statins), medical history and smoking habits were collected from the medical records. All individuals were matched for sex, age and duration of diabetes, which was calculated as time (years) between sample collection and the date of diagnosis. During the visit, blood samples were drawn from each individual. Plasma or serum was separated and stored at -80 °C until further use. Levels of ROMs and BAP were measured as described above.

### Statistical analyses

Differences between groups were assessed by Student’s t test for normally distributed variables and by Mann–Whitney *U* or Wilcoxon tests for non-normally distributed variables. Linear regression analyses were performed to assess the relationship of oxidative stress markers with AIx and PWV. Delta changes in AIx, MDA, ROMs, and SOD were calculated by subtracting the baseline measurement from the measurement obtained after 120 min of the acute hyperglycemic challenge. Differences in oxidative stress parameters with high or low HbA1c and varying DKD status were assessed by one-way ANOVA. A two-tailed *p* value < 0.05 was considered statistically significant. All analyses were performed using GraphPad Prism software (Version 5.04, GraphPad Software, San Diego, CA, USA) and IBM SPSS Statistics 26.0 (IBM Corporation, Somers, NY, USA).

**Ethics approval and consent to participate**

The study protocol is in accordance with the Declaration of Helsinki and approved by the Ethics Committee of the Helsinki and Uusimaa Hospital District (HUS) (491/E5/2006, 238/13/03/00/2015, and HUS-3313-2018, July 3rd 2019). All participants gave their informed written consent.

**Clinical trial number**

Not applicable. This study has not been registered as a clinical trial.

## Results

### Acute hyperglycemic challenge alters circulating oxidant and antioxidant levels

**Fig. 1:**
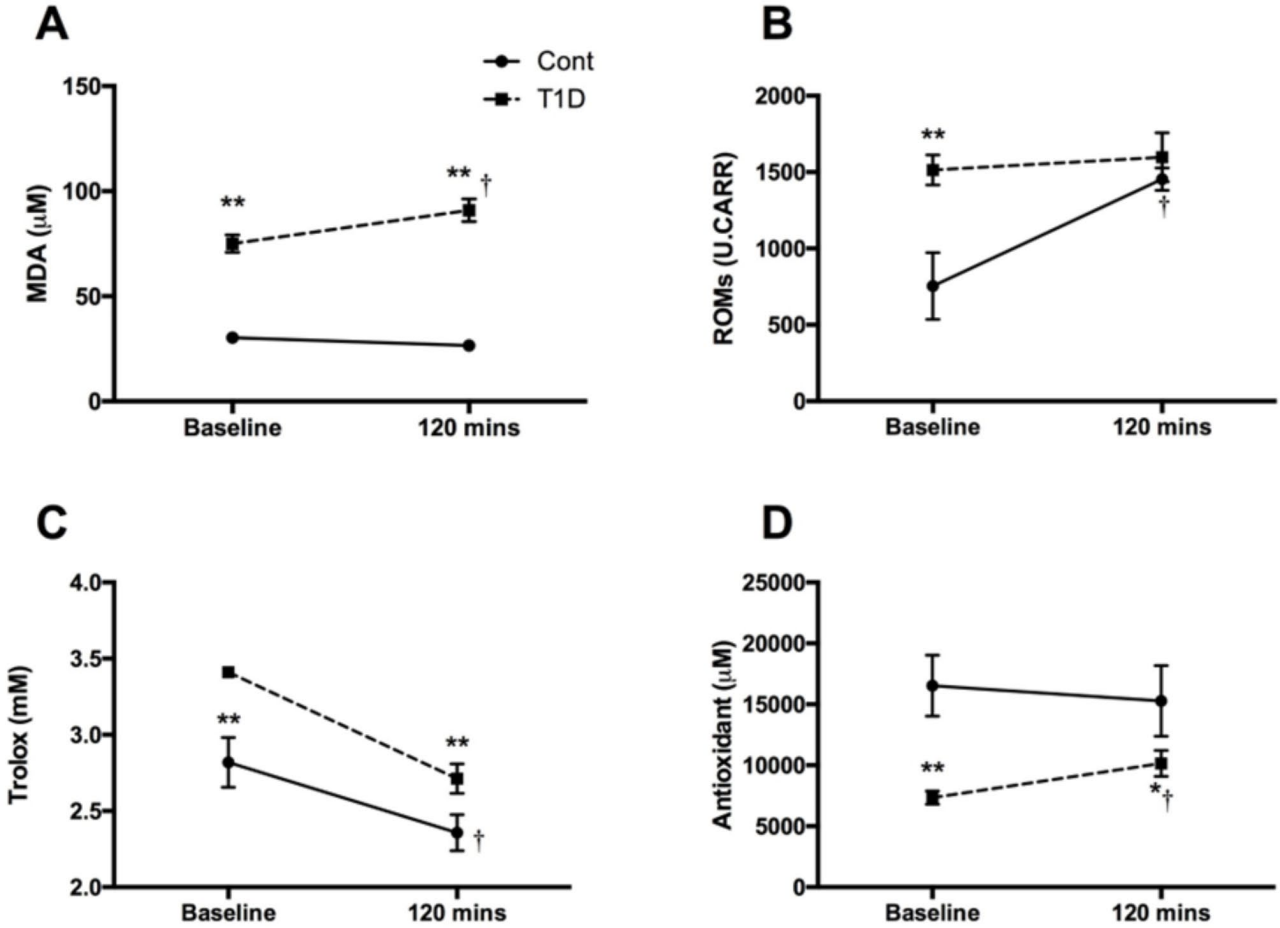
Altered levels of oxidant and antioxidant markers in response to acute hyperglycemia. Plasma levels of MDA, a lipid peroxidation product (A), ROMs (B), total antioxidant capacity by Trolox test (C), and antioxidant levels by biological antioxidant potential test (D) were measured in type 1 diabetes (dotted line) and non-diabetes (solid line) individuals at baseline (normoglycemia) and 120 min after a hyperglycemic challenge. All values are presented as mean ± SEM. **p < 0.01 at baseline; *p < 0.05 compared with control group (120 min); and †*p* < 0.05 compared with the type 1 diabetes group at baseline. MDA, malondialdehyde; ROMs, reactive oxygen metabolites

The individuals with type 1 diabetes had an average age of 25.9 ± 5.6 years with diabetes for an average duration of 9.5 ± 4.4 years. The non-diabetic control group had an average age of 25.4 ± 1.4 years. Additionally, the HbA1c concentrations were significantly higher in the type 1 diabetes group compared to the non-diabetic controls (7.4 ± 0.9% vs. 5.2 ± 0.3%, *P* < 0.01) [6]. Levels of MDA (*p* < 0.01, Fig. 1A) and ROMs (*p* < 0.01, Fig. 1B) products were elevated in type 1 diabetes individuals with normal AER compared to non-diabetic controls at baseline. Following an acute hyperglycemic challenge, individuals with type 1 diabetes showed a further increase in MDA levels (*p* < 0.05) and a sustained elevation of ROMs. In contrast, non-diabetic subjects did not show any difference in MDA levels (Fig. 1A) in response to the challenge, however demonstrated a noticeable increase in ROM levels (*p* < 0.05, Fig. 1B). Plasma antioxidant capacity was measured using the Trolox test and the BAP test. At baseline, type 1 diabetic individuals showed an increased TAC (*p* < 0.01, Fig. 1C), but a decreased BAP capacity (*p* < 0.01, Fig. 1D) compared to non-diabetic controls. The acute hyperglycemic challenge impaired TAC in both type 1 diabetes (*p* < 0.01) and in non-diabetic individuals (*p* < 0.05, Fig. 1C) compared to baseline. BAP was increased (*p* < 0.05) after the challenge in type 1 diabetic individuals compared to baseline, whereas no difference was observed in non-diabetic controls (Fig. 1D).

### Plasma oxidative stress and antioxidant levels are associated with arterial stiffness after acute hyperglycemic challenge

**Fig. 2:**
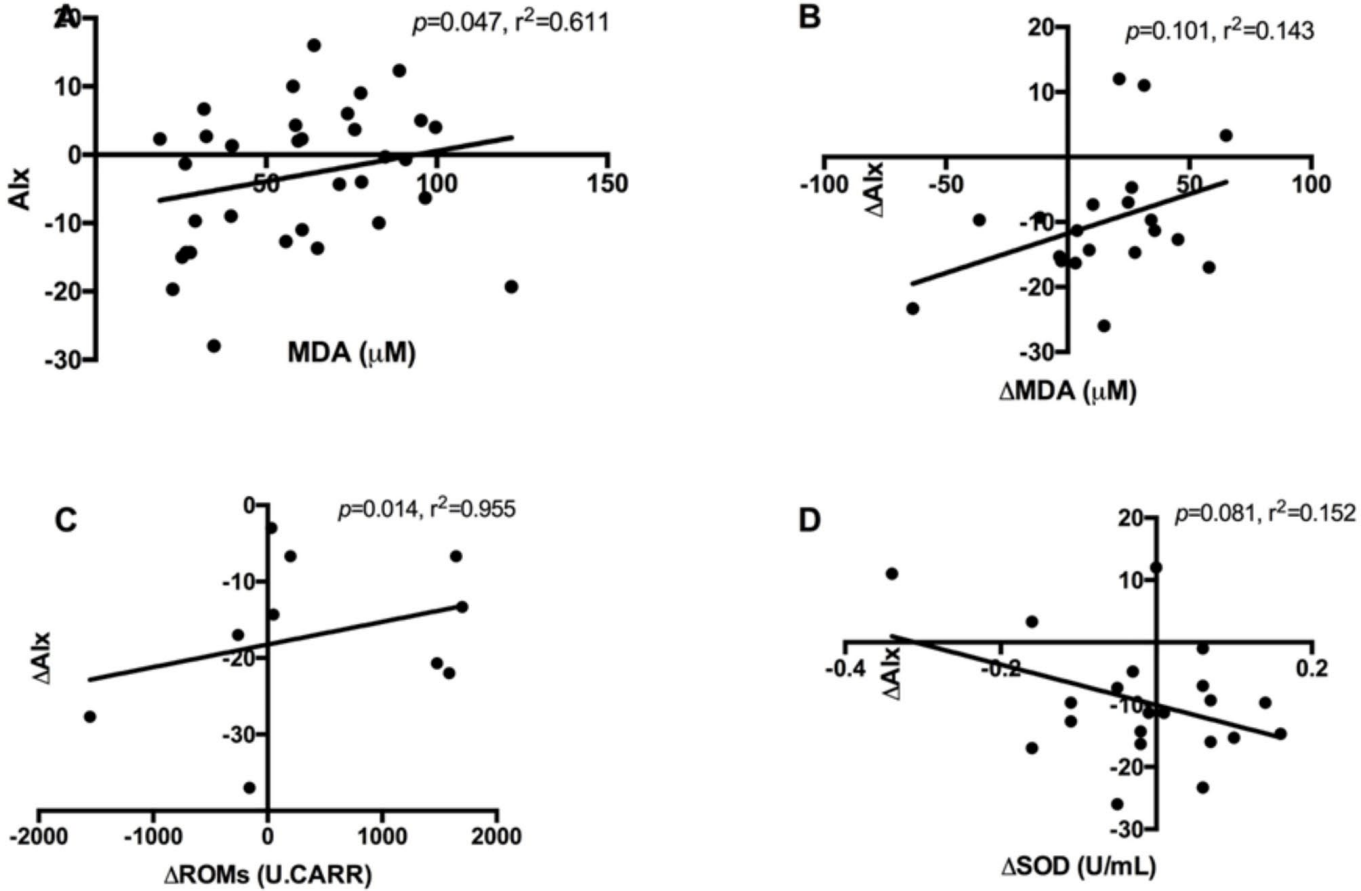
Association between arterial stiffness and oxidative stress markers in type 1 diabetes and non-diabetes individuals. Linear regression analyses were performed to assess the relationship between the arterial stiffness as measured by AIx with plasma levels of MDA, SOD and ROMs. High levels of MDA at baseline (A; diabetes and non-diabetic individuals combined) correlated with increased vascular stiffness (AIx). Following an acute hyperglycemic challenge, change in MDA levels (delta MAD) were positively associated with change in arterial stiffness (delta AIx, B) in individuals with type 1 diabetes. Hyperglycemia-increased ROM levels associated positively with increased AIx (C), whereas increased SOD levels correlated negatively with AIx (D) both in type 1 diabetes and non-diabetic subjects (combined). Delta changes were calculated by subtracting the baseline measurement from that obtained after 120 min of a hyperglycemic challenge. AIx, augmentation index; MDA, malondialdehyde; ROMs, reactive oxygen metabolites; and SOD, superoxide dismutase

In linear regression, at baseline, high circulating MDA levels were positively associated with AIx (r^2^ = 0.611, *p* = 0.05) in a combined analysis of individuals with type 1 diabetes and non-diabetic controls (Fig. 2A). Following an acute hyperglycemic challenge, changes in MDA levels (delta MAD) were positively associated with a change in arterial stiffness (delta AIx) in individuals with type 1 diabetes (r^2^ = 0.143, *p* = 0.101, Fig. 2B), but failed to reach statistical significance. Combined analyses of individuals with type 1 diabetes and non-diabetic controls revealed a positive association between delta ROMs and delta AIx (r^2^ = 0.955, *p* = 0.014, Fig. 2C). Conversely, an elevated SOD concentration was associated with a reduction in AIx (r^2^ = 0.152, *p* = 0.081, Fig. 2D) after the acute hyperglycemic challenge, but the association was non-significant. However, no association was observed between aortic PWV and plasma oxidative stress parameters in individuals with type 1 diabetes or non-diabetic controls (Supplementary Fig. 1). The lack of significant associations may be influenced by the study’s sample size and corresponding statistical power. This suggests that a larger sample size might be required to detect a significant relationship between plasma oxidative stress parameters and arterial stiffness parameters in this population. Our power calculations suggest that this study may be underpowered to identify smaller effect sizes, however on the other hand ensures that the observed associations are more likely to be robust and accurately characterized within this cohort.

### Levels of oxidant and antioxidant in type 1 diabetes individuals with chronic hyperglycemia

**Fig. 3:**
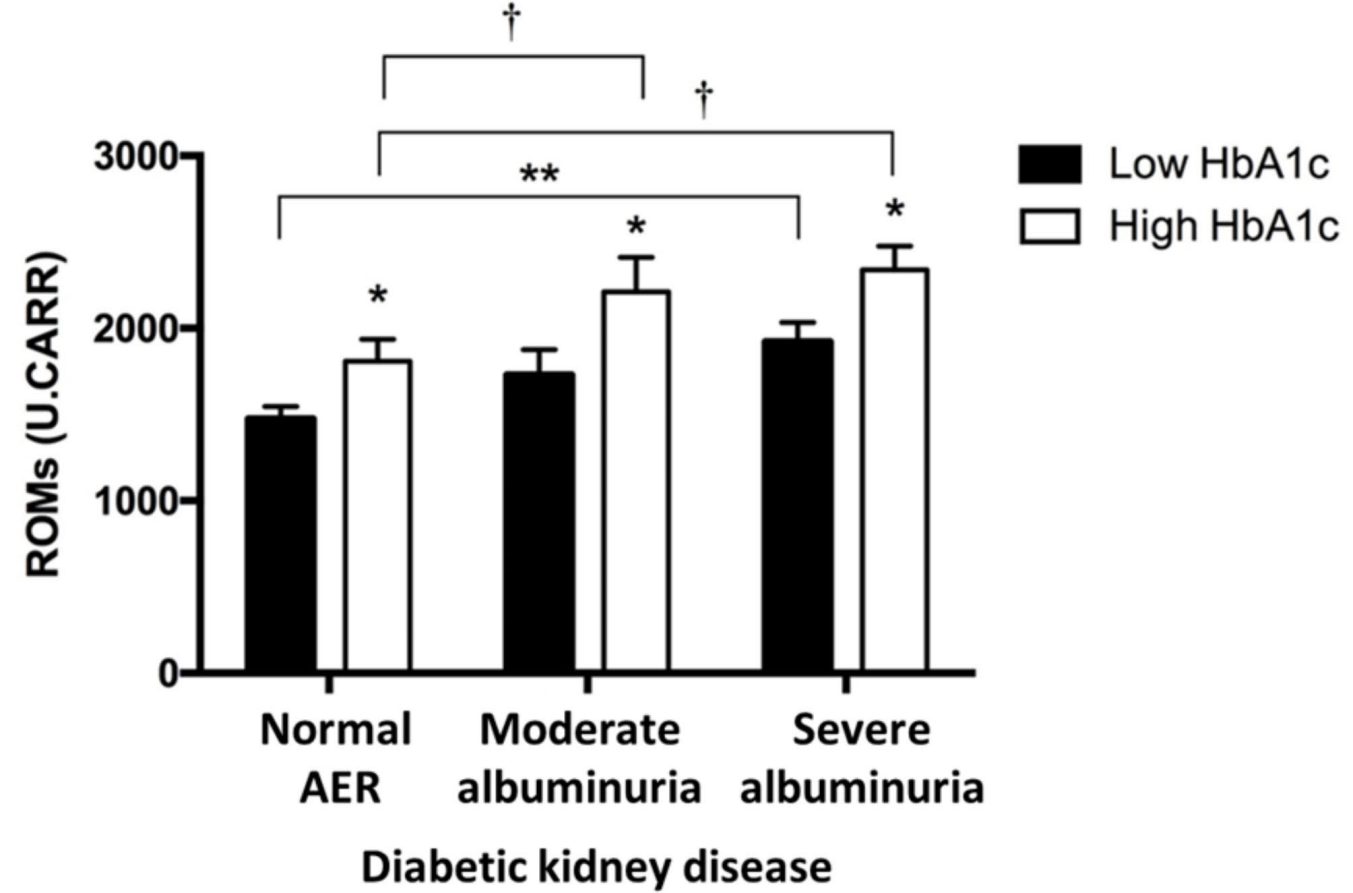
Elevated oxidant levels in type 1 diabetes individuals with varying status of diabetic kidney disease along with low or high HbA1c. Individuals with high HbA1c concentrations (empty bars) show increased plasma reactive oxygen metabolites (ROM) levels compared to individuals with low HbA1c concentrations (filled bars), irrespective of the presence or severity of diabetic kidney disease. Despite comparable HbA1c concentrations, type 1 diabetes individuals with moderate and severe albuminuria showed higher ROM levels when compared to their normal AER counterparts. ROM levels are expressed in mean Carratelli Units (U.CARR.) ± SEM. *p < 0.05 compared with low HbA1c group; **p < 0.01 compared with normal AER, low HbA1c group; and † *p* < 0.01 compared with normal AER, high HbA1c group

Type 1 diabetic individuals with varying status of DKD, showed elevated levels of ROMs in those with high HbA1c compared to their counterparts with low HbA1c (*p* < 0.05). Circulating ROM levels were significantly higher in individuals with moderate (*p* < 0.01) or severe albuminuria (*p* < 0.01) compared to those with normal AER despite comparable HbA1c concentrations (Fig. 3). Modulators of oxidative stress such as age or medication are unable to explain these differences (Supplementary Table 1).

**Fig. 4:**
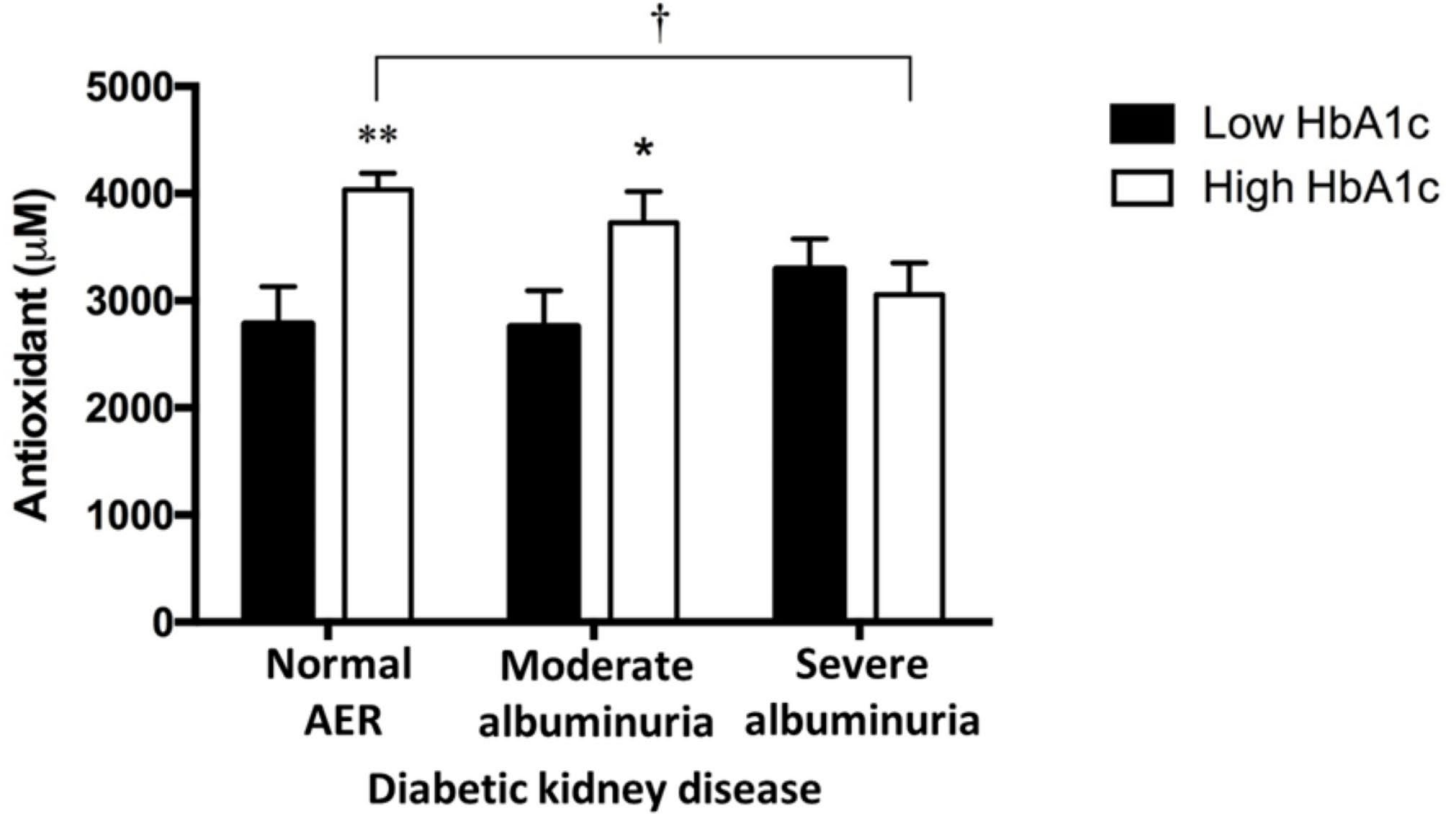
Impaired antioxidant capacity in type 1 diabetes individuals with varying status of diabetic kidney disease along with low or high HbA1c. Type 1 diabetes individuals with high HbA1c (empty bars) and normal AER or moderate albuminuria exhibit elevated plasma antioxidant levels measured as the biological antioxidant potential (BAP) when compared to low HbA1c individuals (filled bars) of comparable diabetic kidney disease status. Conversely, the antioxidant capacity of those with severe albuminuria was no different between high and low HbA1c groups. However, individuals with severe albuminuria display significantly reduced BAP, when compared to individuals with normal AER, despite comparably high HbA1c concentrations. BAP is expressed as the mean [antioxidant] ± SEM. *p < 0.05 compared with low HbA1c group; **p < 0.01 compared with normal AER, low HbA1c group; and †*p* < 0.01 compared with normal AER, high HbA1c group

Individuals with high HbA1c within both the normal AER (*p* < 0.01) group and the moderate albuminuria (*p* < 0.05) group, had higher plasma antioxidant levels compared to those with low HbA1c in the normal AER and moderate albuminuria groups, respectively. Interestingly, type 1 diabetic individuals with severe albuminuria displayed reduced antioxidant levels (*p* < 0.01) compared to those with normal AER of similarly high HbA1c concentrations. Individuals with severe albuminuria did not show any measurable difference in the plasma antioxidant capacity with low or high HbA1c status (Fig. 4).

## Discussion

This study investigated the effect of acute and chronic hyperglycemic exposure on the circulating levels of oxidants and antioxidants in type 1 diabetic individuals with respect to vascular stiffness and DKD. The data showed a clear difference with systemic elevation of oxidative stress parameters in type 1 diabetes compared to non-diabetic subjects at baseline and following an acute hyperglycemic challenge. However, TAC was increased and BAP was reduced in type 1 diabetic individuals compared to non-diabetic controls at baseline. This trend was reversed after an acute hyperglycemic challenge with reduced TAC and increased BAP in type 1 diabetic individuals compared to their respective baseline levels. The change in ROMs was associated with increased vascular stiffness (delta AIx). In chronic hyperglycemia, striking imbalances between oxidative stress (ROMs) and antioxidant (BAP) parameters were observed in diabetic subjects with high HbA1c and different DKD status. These data are in line with increasing experimental evidence from mouse models of diabetes-accelerated vascular and kidney disease demonstrating a central role for oxidative stress in mediating the noxious effects of hyperglycemia on the vasculature (2; 16). Here, we provide new evidence supporting the potential clinical utility of oxidative stress biomarkers for the identification and assessment of vascular and kidney dysfunction in type 1 diabetes.

Lipid peroxidation causes deleterious biological effects including NF-kB-mediated inflammation and macromolecule modification, known to contribute to arterial stiffness [18] and kidney disease [19] in diabetes. Reduced antioxidants induce significant damage to tissues due to more lipid peroxidation production in chronic diabetic conditions independent of glycemic control [20]. An acute hyperglycemic challenge causes passive influx of potassium (86Rb+) into erythrocytes accompanying elevated lipid peroxidation, this phenomenon being the earliest change that occurs in response to a 2 h glucose load in subjects with normal glucose tolerance [21, 22]. Similarly, postprandial MDA concentrations were increased to a greater extent following the ingestion of glucose compared to fructose in healthy men during a 3-h postprandial period [23]. Consistent with these findings the present study reports increased levels of the lipid peroxidation product MDA after an acute hyperglycemic challenge in type 1 diabetic individuals. Acute changes in cellular glucose metabolism lead to an imbalance in the previously steady levels of ROS to promote more free radical generation and decrease antioxidant defense systems [24]. Similarly in the present study the exposure to hyperglycemia remarkably increased hydroperoxide generation as estimated by ROM levels in individuals with type 1 diabetes compared to non-diabetic subjects at baseline. Interestingly, no change was observed in ROM levels after the acute hyperglycemic challenge in type 1 diabetic individuals. This finding aligns with previous study on cells showing a dynamic relationship between ROS production and mitochondrial morphology, where high glucose exposure neither increases ROS nor mitochondrial fragmentation [25]. This observation may suggest a compensatory mechanism in response to metabolic stress, possibly contributing to the sustained ROM levels observed in type 1 diabetic individuals. More accumulation and decreased clearance of ROS leads to enhanced MDA formation and together these markers of oxidative stress serve as important pathological indicators to confirm the severity of oxidant status during a rapid glucose change [26]. These findings prove that oxidative stress is an early event particularly responsive to additional hyperglycemic insults and likely represents an important pathway of oxidant-mediated organ damage in uncontrolled diabetes.

In addition to enhanced oxidant formation, hyperglycemia alters cytoprotective systems that scavenge/inactivate ROS. Previously, our group showed that circulating SOD levels are significantly higher in type 1 diabetic individuals compared to healthy volunteers [6]. Similarly, in the present study, individuals with type 1 diabetes showed an increased capacity to prevent oxidation by free radicals (Trolox test) but a decreased capacity to reduce ferric ions (BAP test) compared to non-diabetic controls at baseline. The lack of correlation between these two assays has previously been reported; indeed, they use different technologies to provide information on distinct antioxidant systems [27]. Of note, our optimization of the BAP test to a high-throughput 96-well plate format resulted in higher values than have been previously reported; however, the sample absorbance values were comparable to the internal calibrator as well as the low and high serum controls provided by the manufacturer (*data not shown*). Additionally, we conducted a power analysis with an alpha level of 0.05 to determine the sample size needed to detect significant differences in antioxidant capacity between type 1 diabetic individuals and non-diabetic controls. The analysis indicated a power of 99% to detect differences in total antioxidant capacity (TAC) with a partial eta squared of 0.371 (*n* = 35). However, the power was only 12% for detecting differences in individual’s antioxidants levels with a partial eta squared of 0.020 (*n* = 33). This low power may explain the lack of significant changes in antioxidants detected by the BAP test in non-diabetic controls.

The effects of hyperglycemia on antioxidant defence systems are not straightforward and are likely dependent on the enzyme in question. Therefore, multiple assays are required to assess antioxidant parameters. In type 2 diabetic individuals, the activities of two antioxidant enzymes, catalase and glutathione peroxidase, have been reported to be differentially regulated when compared to controls [28]. Exposure of rat aorta to high glucose reduced SOD and GSH activity and increased MDA levels confirming redox imbalance upon hyperglycemic stress [29]. Consistent with these findings, a previous study showed a marked reduction in antioxidant concentrations up to 120 min after an oral glucose tolerance test (OGTT) [30] in individuals with suspected diabetes. These results are similar to the present findings of reduced TAC after an acute hyperglycaemic challenge. Collectively, these findings suggest that oxidant production driven by hyperglycaemia triggers compensatory antioxidant responses to prevent further radical-mediated damage; however, excessive ROS burden stimulated by acute hyperglycaemic exposure overwhelms the protective antioxidant capacity leading to unrestrained lipid peroxide generation [31].

Hyperglycaemia-induced oxidative stress is a well-established pathological mediator of diabetes-associated vascular dysfunction and disease [32]. Chronic exposure to hyperglycemia induces damage to the micro- or macrovasculature, and in a recent report a rapid blood glucose change was associated with increased arterial stiffness in normal glucose-tolerant (NGT) subjects [33]. We have previously demonstrated a marked increase in vascular stiffness at baseline and following a hyperglycemic challenge in individuals with uncomplicated type 1 diabetes compared to non-diabetic controls [34]. This finding was extended in the present study by showing a positive association between arterial stiffness and plasma oxidant levels. Combined analyses of type 1 diabetic and non-diabetic individuals showed that a hyperglycemia-mediated increase in plasma SOD was associated with a reduction in AIx. Consistent with these findings, Gomez-Marcos et al. [35] detected negative correlations between SOD and several parameters of vascular stiffness in hypertensive individuals with type 2 diabetes. Interestingly, we did not observe any significant association between plasma oxidative stress parameters and PWV. In regard, PWV reflects the long-term structural changes in the large arterial wall, whereas AIx reflects the short-term changes influenced by both large and small arteries [36]. An acute hyperglycemic challenge may cause acute alterations in the oxidant-antioxidants balance, potentially impacting AIx more prominently than PWV. Oxidative stress markers measured, such as lipid peroxidation by MDA, and ROM are more closely linked to endothelial dysfunction and acute changes, likely influencing AIx rather than the structural integrity and stiffness of large arteries measured by PWV. Moreover, variability in the individual’s response and study power could also potentially mask the associations between oxidative stress parameters and PWV in this cohort. These findings require further investigations in larger cohorts with long-term studies to investigate the relationships between oxidative stress and vascular function.

The Diabetes Control and Complications Trial (DCCT) demonstrated that intensive insulin therapy aimed at reducing hyperglycemic excursions delays the onset and retards the progression of microvascular complications [37]. In this study, we explored the potential differences in oxidant/antioxidant levels between diabetic individuals with good and poor glycemic control (low/ high HbA1c values) and distinct kidney function presentations. Glycemic variability predicts the risk of vascular complications, while HbA1c levels are directly associated with the mortality risk of individuals with diabetes [38, 39]. High HbA1c or intracellular glucose-induced oxidative stress and imbalances in antioxidants contribute to endothelial impairment and vascular damage. A recent report confirms that treatment of diabetic plasma with high HbA1c (≥ 10% or 86 mmol/mol) on normal semen samples affected all sperm motility parameters and increased oxidative stress markers [40]. Similarly, another study showed a mechanistic connection between oxidative stress and DKD, where AOPP, a trigger of oxidative stress increases FOXO3a protein levels by inhibiting their autophagic degradation in a ROS/mTOR-dependent manner and causes podocyte injury in DKD [40]. Consistent with these findings our study showed that type 1 diabetic individuals with high HbA1c display increased levels of ROMs and reduced antioxidants with advancing DKD status. Antioxidant systems protect against continuously generated ROS and maintain redox balance. During chronic hyperglycemia, a compensatory mechanism is activated via generating more antioxidants in order to normalize excessive oxidative stress [42], supporting the present finding of higher antioxidant potential with high HbA1c in type 1 diabetes individuals with normal and moderate albuminuria. Additionally, elevated antioxidants accompanying high HbA1c in response to increased oxidative burden except for those individuals with severe albuminuria ultimately increases their vulnerability to ROS-mediated damage that accelerates progression to end-stage kidney disease. This study indicates that a disturbance in oxidant/antioxidant balance is the adverse outcome of acute or chronic hyperglycemic events and could serve as an early prediction marker for disease progression.

Although, the study has a relatively small sample size, it is of note that it is the first study examining the effect of both acute and chronic hyperglycemia on oxidative stress in adults with type 1 diabetes. It’s also worth mentioning that elevated insulin concentrations outside the normal range can reduce arterial stiffness. Thus, any vascular effects from hyperglycemia could potentially be influenced by increased insulin concentrations. In the healthy non-diabetic controls, the insulin secretion was inhibited by using somatostatin to eliminate the insulin influence. We did not analyze the influence of other hormones such as glucagon, catecholamines, cortisol, growth hormone, and prolactin on observed associations. On the other hand, the study benefits from a well-characterized cohort of Finnish participants with type 1 diabetes, and strict exclusion criteria such as non-smoking status, absence of diabetic complications, and no medications other than insulin use.

Taken together, this study highlights the complex interplay between oxidant and antioxidant systems being activated/inactivated by chronic and acute exposure to hyperglycemia. Chronic exposure to hyperglycemia alters oxidant and antioxidant responses to subsequent acute hyperglycemic challenges, which is associated with increased vascular stiffness. Poor glycemic control in type 1 diabetic individuals is associated with increased systemic oxidative burden, which is further exacerbated in patients with DKD. Overall, glucose load or excessive glycemic change could be toxic events to cells, which impairs cellular function by increasing ROS production. In the search for novel biomarkers for early disease detection and improved prognosis, the assessment of oxidant-antioxidant status may provide added value to HbA1c and arterial stiffness in identifying ‘high risk’ groups for DKD.

## Supplementary Information

Supplemental Figure 1: No correlation between circulating MDA, SOD and ROM levels and PWV in type 1 diabetes and non-diabetic patients. Linear regression analyses were performed to assess the relationship between stiffness as estimated by aortic PWV (A-D) and brachial PWV (E-H) with plasma concentration levels of MDA, SOD and ROMs. Delta change values were calculated by subtracting the baseline measurement from that obtained after 120 minutes of hyperglycemia. MDA, malondialdehyde; PWV, pulse-wave velocity; ROMs, reactive oxygen metabolites; and SOD, superoxide dismutase. Supplementary Material 1.

Supplementary Material 2

## Data Availability

No datasets were generated or analysed during the current study.

## Abbreviations

Aix: Augmentation index
BAP: Biological antioxidant potential
CVD: Cardiovascular disease
DKD: Diabetic kidney disease
ESKD: End-stage kidney disease
HG: Hyperglycemia
MDA: Malondialdehyde
ND: Non-diabetic
NOX: NADPH oxidase
PWV: Pulse wave velocity
ROMs: Reactive oxygen metabolites
ROS: Reactive oxygen species
SOD: Superoxide dismutase
TBARS: Thiobarbituric acid reactive substances
UAER: Urinary albumin excretion rate
TAC: Total antioxidant capacity

## Declarations

### Clinical trial number

Not applicable. This study has not been registered as a clinical trial.
