# Supplementary material for: Altered oxidant and antioxidant levels are associated with vascular stiffness and diabetic kidney disease in type 1 diabetes after exposure to acute and chronic hyperglycemia
Source: Cardiovasc Diabetol. 2024 Sep 28;23:350. doi: 10.1186/s12933-024-02427-4 (PMC11439198; doi:10.1186/s12933-024-02427-4)
Supplement: Supplementary file 1 — Supplemental Figure 1: No correlation between circulating MDA, SOD and ROM levels and PWV in type 1 diabetes and non-diabetic patients. Linear regression analyses were performed to assess the relationship between stiffness as estimated by aortic PWV (A-D) and brachial PWV (E-H) with plasma concentration levels of MDA, SOD and ROMs. Delta change values were calculated by subtracting the baseline measurement from that obtained after 120 minutes of hyperglycemia. MDA, malondialdehyde; PWV, pulse-wave velocity; ROMs, reactive oxygen metabolites; and SOD, superoxide dismutase. Supplementary Material 1. [file 12933_2024_2427_MOESM1_ESM.docx]

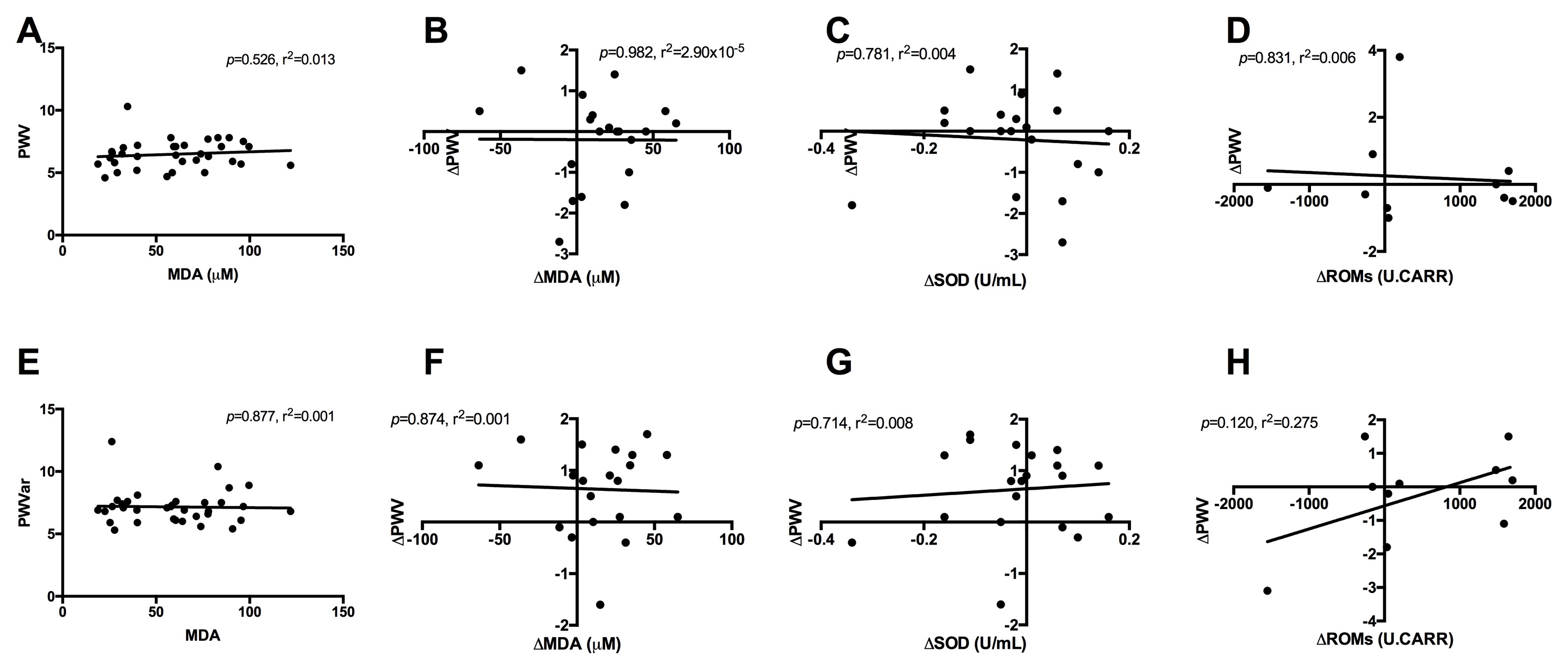


**Supplemental Figure 1: No correlation between circulating MDA, SOD and ROM levels and PWV in type 1 diabetes and non-diabetic patients.** Linear regression analyses were performed to assess the relationship between stiffness as estimated by aortic PWV (A-D) and brachial PWV (E-H) with plasma concentration levels of MDA, SOD and ROMs. Delta change values were calculated by subtracting the baseline measurement from that obtained after 120 minutes of hyperglycemia. MDA, malondialdehyde; PWV, pulse-wave velocity; ROMs, reactive oxygen metabolites; and SOD, superoxide dismutase.
