# Supplementary material for: Altered oxidant and antioxidant levels are associated with vascular stiffness and diabetic kidney disease in type 1 diabetes after exposure to acute and chronic hyperglycemia
Source: Cardiovasc Diabetol. 2024 Sep 28;23:350. doi: 10.1186/s12933-024-02427-4 (PMC11439198; doi:10.1186/s12933-024-02427-4)
Supplement: Supplementary file 2 — Supplementary Material 2 [file 12933_2024_2427_MOESM2_ESM.docx]

**Supplemental Table 1.** Clinical characteristics of study participants based on varying status of diabetic kidney disease along with low or high HbA1c.

|  | Normal AER/Low | Normal AER/High | Moderate albuminuria /Low | Moderate albuminuria /High | Severe albuminuria/Low | Severe albuminuria/High |
| --- | --- | --- | --- | --- | --- | --- |
| Male/female | 10/10 | 7/13 | 10/10 | 8/10 | 10/10 | 8/12 |
| Age (years) | 49.1 ± 14.0 | 42.1 ± 14.3 | 52.8 ± 12.0 | 37.6 ± 11.3 | 50.5 ± 10.4 | 44.4 ± 10.2 |
| Duration of diabetes (years) | 31.0 ± 13.4 | 25.9 ± 7.8 | 36.3 ± 12.5 | 28.0 ± 11.7 | 39.4 ± 10.3 | 33.6 ± 9.2 |
| BMI (kg/m²) | 25.6 ± 2.7 | 24.7 ± 2.9 | 26.6 ± 5.0 | 27.7 ± 4.0 | 27.2 ± 5.5 | 25.6 ± 5.0 |
| Systolic BP (mmHg) | 131 ± 14 | 135 ± 20 | 137 ± 15 | 138 ± 17 | 150 ± 22 | 140 ± 23 |
| Diastolic BP (mmHg) | 76 ± 8 | 80 ± 7 | 73 ± 10 | 78 ± 11 | 79 ± 11 | 80 ± 10 |
| ACEi/ARBs (%) | 15.0 | 15.8 | 60.0 | 88.9 | 95.0 | 75.0 |
| Mean HbA1c (%) | 6.2 ± 0.5 | 10.7 ± 0.8 | 6.9 ± 0.5 | 10.8 ± 0.6 | 6.9 ± 0.4 | 11.0 ± 1.0 |
| Mean HbA1c (mmol/mol) | 44 ± 6 | 93 ± 8 | 52 ± 6 | 95 ± 6 | 52 ± 4 | 97 ± 11 |
| N of measurements/5 years | 12 ± 5 | 13 ± 5 | 12 ± 5 | 15 ± 5 | 17 ± 7 | 17 ± 6 |
| Total cholesterol (mmol/l) | 4.86 ± 0.90 | 4.77 ± 0.75 | 4.98 ± 0.96 | 4.85 ± 0.97 | 4.48 ± 0.96 | 4.70 ± 1.05 |
| HDL-cholesterol (mmol/l) | 1.71 ± 0.46 | 1.54 ± 0.36 | 1.83 ± 0.58 | 1.42 ± 0.56 | 1.47 ± 0.31 | 1.42 ± 0.42 |
| Triglycerides (mmol/l) | 0.76 (0.62 - 1.03) | 1.18 (1.05 - 1.46) | 0.87 (0.63 - 1.37) | 1.63 (1.02 – 2.15) | 1.36 (0.93 – 1.76) | 1.79 (1.14 -2.37) |
| Statins (%) | 20.0 | 15.8 | 45.0 | 44.4 | 65.0 | 63.2 |
| eGFR (ml/min/1.73m^2^) | 93 (80 – 108) | 113 (103 – 116) | 92 (72 – 103) | 99 (87 – 120) | 44 (29 – 80) | 48 (32 – 72) |

Data are presented as means ± SD or median (IQR) or proportions. Low and High denotes groups with high and low HbA1c. AER: albumin excretion rate; BMI: body mass index; BP: blood pressure; ACEi: Angiotensin-Converting Enzyme Inhibitor; ARBs: Angiotensin receptor blockers; HbA1c: glycated haemoglobin; HDL: high-density lipoproteins; eGFR: estimated glomerular filtration rate.

**APPENDIX**

**The Finnish Diabetic Nephropathy Study Centers**

Anjalankoski Health Center S.Koivula, T.Uggeldahl

Central Finland Central Hospital, Jyväskylä T.Forslund, A.Halonen, A.Koistinen, P.Koskiaho,

M.Laukkanen, J.Saltevo, M.Tiihonen

Central Hospital of Åland Islands, Mariehamn M.Forsen, H.Granlund, A.-C.Jonsson, B.Nyroos

Central Hospital of Kanta-Häme, Hämeenlinna P.Kinnunen, A.Orvola, T.Salonen, A.Vähänen

Central Hospital of Kymenlaakso, Kotka R.Paldanius, M.Riihelä, L.Ryysy

Central Hospital of Länsi-Pohja, Kemi H.Laukkanen, P.Nyländen, A.Sademies

Central Ostrobothnian Hospital District, Kokkola S.Anderson, B.Asplund, U.Byskata, P.Liedes,

M.Kuusela, T.Virkkala

City of Espoo Health Center:

Espoonlahti A.Nikkola, E.Ritola

Tapiola M.Niska, H.Saarinen

Samaria E.Oukko-Ruponen, T.Virtanen

Viherlaakso A.Lyytinen

City of Helsinki Health Center:

Puistola H.Kari, T.Simonen

Suutarila A.Kaprio, J.Kärkkäinen, B.Rantaeskola

Töölö P.Kääriäinen, J.Haaga, A-L.Pietiläinen

City of Hyvinkää Health Center S.Klemetti, T.Nyandoto, E.Rontu, S.Satuli-Autere

City of Vantaa Health Center:

Korso R.Toivonen, H.Virtanen

Länsimäki R.Ahonen, M.Ivaska-Suomela, A.Jauhiainen

Martinlaakso M.Laine, T.Pellonpää, R.Puranen

Myyrmäki A.Airas, J.Laakso, K.Rautavaara

Rekola M.Erola, E.Jatkola

Tikkurila R.Lönnblad, A.Malm, J.Mäkelä, E.Rautamo

Heinola Health Center P.Hentunen, J.Lagerstam

Helsinki University Central Hospital, Department of

Medicine, Division of Nephrology A.Ahola, M.Feodoroff, D.Gordin, O.Heikkilä, K.Hietala, M.Korolainen J.Kytö, S.Lindh, K.Pettersson-Fernholm, A.Sandelin, L.Thorn, J.Tuomikangas, T.Vesisenaho, J.Wadén

Herttoniemi Hospital, Helsinki V.Sipilä

Hospital of Lounais-Häme, Forssa T.Kalliomäki, J.Koskelainen, R.Nikkanen,

N.Savolainen, H.Sulonen, E.Valtonen

Hyvinkää Hospital L. Norvio, A. Hämäläinen

Iisalmi Hospital E.Toivanen

Jokilaakso Hospital, Jämsä A.Parta, I.Pirttiniemi

Jorvi Hospital, Helsinki University Central Hospital S.Aranko, S.Ervasti, R.Kauppinen-Mäkelin,

A.Kuusisto, T.Leppälä, K.Nikkilä, L.Pekkonen

Jyväskylä Health Center, Kyllö K.Nuorva, M.Tiihonen

Kainuu Central Hospital, Kajaani S.Jokelainen, K.Kananen, M.Karjalainen, P.Kemppainen, A-M.Mankinen, A.Reponen

M.Sankari

Kerava Health Center H.Stuckey, P.Suominen

Kirkkonummi Health Center A.Lappalainen, M.Liimatainen, J.Santaholma

Kivelä Hospital, Helsinki A.Aimolahti, E.Huovinen

Koskela Hospital, Helsinki V.Ilkka, M.Lehtimäki

Kotka Health Center E.Pälikkö-Kontinen, A.Vanhanen

Kouvola Health Center E.Koskinen, T.Siitonen

Kuopio University Hospital E.Huttunen, R.Ikäheimo, P.Karhapää, P.Kekäläinen,

M.Laakso, T.Lakka, E.Lampainen, L.Moilanen,

L.Niskanen, U.Tuovinen, I.Vauhkonen, E.Voutilainen

Kuusamo Health Center T.Kääriäinen, E.Isopoussu

Kuusankoski Hospital E.Kilkki, I.Koskinen, L.Riihelä

Laakso Hospital, Helsinki T.Meriläinen, P.Poukka, R.Savolainen, N.Uhlenius

Lahti City Hospital A.Mäkelä, M.Tanner

Lapland Central Hospital, Rovaniemi L.Hyvärinen, K.Lampela, S.Pöykkö, T.Rompasaari, S.Severinkangas, T.Tulokas

Lappeenranta Health Center P. Erola, L. Härkönen, P.Linkola, I.Pulli, E.Repo

Lohja Hospital T.Granlund, K.Hietanen, M.Porrassalmi, M.Saari, T.Salonen, M.Tiikkainen,

Länsi-Uusimaa Hospital, Tammisaari I.-M.Jousmaa, J.Rinne

Loimaa Health Center A.Mäkelä, P.Eloranta

Malmi Hospital, Helsinki H.Lanki, S.Moilanen, M.Tilly-Kiesi

Mikkeli Central Hospital A.Gynther, R.Manninen, P.Nironen, M.Salminen,

T.Vänttinen

Mänttä Regional Hospital I.Pirttiniemi, A-M.Hänninen

North Karelian Hospital, Joensuu U-M.Henttula, P.Kekäläinen, M.Pietarinen,

A.Rissanen, M.Voutilainen

Nurmijärvi Health Center A.Burgos, K.Urtamo

Oulaskangas Hospital, Oulainen E.Jokelainen, P-L.Jylkkä, E.Kaarlela, J.Vuolaspuro

Oulu Health Center L.Hiltunen, R.Häkkinen, S.Keinänen-Kiukaanniemi

Oulu University Hospital R.Ikäheimo

Päijät-Häme Central Hospital H.Haapamäki, A.Helanterä, S.Hämäläinen,

V.Ilvesmäki, H.Miettinen

Palokka Health Center P.Sopanen, L.Welling

Pieksämäki Hospital V.Sevtsenko, M.Tamminen

Pietarsaari Hospital M-L.Holmbäck, B.Isomaa, L.Sarelin

Pori City Hospital P.Ahonen, P.Merisalo, E.Muurinen, K.Sävelä

Porvoo Hospital M.Kallio, B.Rask, S.Rämö

Raahe Hospital A.Holma, M.Honkala, A.Tuomivaara, R.Vainionpää

Rauma Hospital K.Laine, K.Saarinen, T.Salminen

Riihimäki Hospital P.Aalto, E.Immonen, L.Juurinen

Salo Hospital A.Alanko, J.Lapinleimu, P.Rautio, M.Virtanen

Satakunta Central Hospital, Pori M.Asola, M.Juhola, P.Kunelius, M.-L.Lahdenmäki,

P.Pääkkönen, M.Rautavirta

Savonlinna Central Hospital T.Pulli, P.Sallinen, M.Taskinen, E.Tolvanen, T.Tuominen

H.Valtonen, A.Vartia, S-L.Viitanen

Seinäjoki Central Hospital O.Antila, E.Korpi-Hyövälti, T.Latvala, E.Leijala, T.Leikkari, M.Punkari N.Rantamäki, H.Vähävuori

South Karelia Central Hospital, Lappeenranta T.Ensala, E.Hussi, R.Härkönen, U.Nyholm, J.Toivanen

Tampere Health Center A.Vaden, P.Alarotu, E.Kujansuu, H.Kirkkopelto-Jokinen,

M.Helin, S.Gummerus, L.Calonius, T.Niskanen, T.Kaitala,

T.Vatanen

Tampere University Hospital I.Ala-Houhala, R.Kannisto, T.Kuningas, P.Lampinen, M.Määttä,

H.Oksala, T.Oksanen, A.Putila, H.Saha, K.Salonen, H.Tauriainen,

S.Tulokas

Tiirismaa Health Center, Hollola T.Kivelä, L.Petlin, L.Savolainen

Turku Health Center A.Artukka, I.Hämäläinen, L.Lehtinen, E.Pyysalo, H.Virtamo, M.Viinikkala, M.Vähätalo

Turku University Central Hospital K.Breitholz, R.Eskola, K.Metsärinne, U.Pietilä,

P.Saarinen, R.Tuominen, S.Äyräpää

Vaajakoski Health Center K.Mäkinen, P.Sopanen

Valkeakoski Regional Hospital S.Ojanen, E.Valtonen, H.Ylönen, M.Rautiainen,

T.Immonen

Vammala Regional Hospital I.Isomäki, R.Kroneld, L.Mustaniemi, M.Tapiolinna-Mäkelä

Vasa Central Hospital S.Bergkulla, U.Hautamäki, V-A.Myllyniemi, I.Rusk
